# Photosynthetic conversion of CO_2_ to farnesyl diphosphate-derived phytochemicals (amorpha-4,11-diene and squalene) by engineered cyanobacteria

**DOI:** 10.1186/s13068-016-0617-8

**Published:** 2016-09-22

**Authors:** Sun Young Choi, Hyun Jeong Lee, Jaeyeon Choi, Jiye Kim, Sang Jun Sim, Youngsoon Um, Yunje Kim, Taek Soon Lee, Jay D. Keasling, Han Min Woo

**Affiliations:** 1 Clean Energy Research Center, Korea Institute of Science and Technology, Hwarangro 14-gil 5, Seongbuk-gu, Seoul, 02792 Republic of Korea; 2 Green School (Graduate School of Energy and Environment), Korea University, 145 Anam-ro, Seongbuk-gu, Seoul, 02841 Republic of Korea; 3 Department of Chemistry, Korea University, 145 Anam-ro, Seongbuk-gu, Seoul, 02841 Republic of Korea; 4 Department of Chemical and Biological Engineering, Korea University, 145 Anam-ro, Seongbuk-gu, Seoul, 02841 Republic of Korea; 5 Joint BioEnergy Institute, 5885 Hollis Street, Emeryville, CA 94608 USA; 6 Biological Systems & Engineering Division, Lawrence Berkeley National Laboratory, Berkeley, CA 94720 USA; 7 Department of Bioengineering, University of California, Berkeley, CA 94720 USA; 8 Department of Chemical and Biomolecular Engineering, University of California, Berkeley, CA 94720 USA; 9 Department of Food Science and Biotechnology, Sungkyunkwan University (SKKU), 2066 Seobu-ro, Jangan-gu, Suwon, 16419 Republic of Korea

**Keywords:** Metabolic engineering, Cyanobacteria, Synthetic biology, Isoprenoids

## Abstract

**Background:**

Metabolic engineering of cyanobacteria has enabled photosynthetic conversion of CO_2_ to value-added chemicals as bio-solar cell factories. However, the production levels of isoprenoids in engineered cyanobacteria were quite low, compared to other microbial hosts. Therefore, modular optimization of multiple gene expressions for metabolic engineering of cyanobacteria is required for the production of farnesyl diphosphate-derived isoprenoids from CO_2_.

**Results:**

Here, we engineered *Synechococcus elongatus* PCC 7942 with modular metabolic pathways consisting of the methylerythritol phosphate pathway enzymes and the amorphadiene synthase for production of amorpha-4,11-diene, resulting in significantly increased levels (23-fold) of amorpha-4,11-diene (19.8 mg/L) in the best strain relative to a parental strain. Replacing amorphadiene synthase with squalene synthase led to the synthesis of a high amount of squalene (4.98 mg/L/OD_730_). Overexpression of farnesyl diphosphate synthase is the most critical factor for the significant production, whereas overexpression of 1-deoxy-d-xylulose 5-phosphate reductase is detrimental to the cell growth and the production. Additionally, the cyanobacterial growth inhibition was alleviated by expressing a terpene synthase in *S. elongatus* PCC 7942 strain with the optimized MEP pathway only (SeHL33).

**Conclusions:**

This is the first demonstration of photosynthetic production of amorpha-4,11-diene from CO_2_ in cyanobacteria and production of squalene in *S. elongatus* PCC 7942. Our optimized modular OverMEP strain (SeHL33) with either co-expression of ADS or SQS demonstrated the highest production levels of amorpha-4,11-diene and squalene, which could expand the list of farnesyl diphosphate-derived isoprenoids from CO_2_ as bio-solar cell factories.

**Electronic supplementary material:**

The online version of this article (doi:10.1186/s13068-016-0617-8) contains supplementary material, which is available to authorized users.

## Background

Terpenoids extracted from plants are widely used as flavors, fragrances, and medicines. Because these compounds are naturally produced in small quantities, plant metabolic engineering has been applied in different compartments for the overproduction of target terpenoids: mono- and diterpenes and carotenoids in the plastids and sesqui- and triterpenes in the cytosol [1, 2]. The methylerythritol phosphate (MEP) pathway has been engineered to enhance the production of volatilized terpenoids [i.e., geranyl diphosphate (GPP)-derived monoterpene] such as linalool [3], geraniol [4], and limonene [5] by overexpressing the corresponding terpene synthases with engineered promoters in the plastids of carnation, tomato, and tobacco, respectively. On the other hand, metabolic engineering studies for higher production of sesquiterpene (i.e., artemisinin) has been extensively performed in the cytosol of *Artemisia annua* L. by overexpressing the mevalonate pathway enzymes [6], amorpha-4,11-diene synthase (ADS) [7], transcriptional factors to increase the precursor supply and repressing enzymes that compete for the precursor using the RNA interference-based suppression [8]. Also, synthetic biology has been employed to produce artemisinin by reconstructing the heterologous metabolic pathway and optimizing the gene expression of ADS and its cognitive enzymes in a non-native fast-growing crop plant (i.e., tobacco) [9] or to produce various farnesyl diphosphate (FPP)-derived chemicals by altering the catalytic functions of terpene cyclases in *A. annua* L. [10].

Heterologous terpenoids including mono- and sesquiterpenes and carotenoids have also been produced in engineered microbes without compartments. For microbial production of carotenoids via the MEP pathway, the strains have been engineered by increasing the accumulation of precursors [11] or optimizing phenotype through high-throughput screening methods [12, 13] with expressing heterologous carotenoid biosynthetic enzymes. Besides, the heterologous mevalonate pathway has been introduced to produce high amounts of heterologous monoterpenes such as limonene and pinene in *Escherichia coli* [14, 15] and sesquiterpenes such as amorpha-4,11-diene and farnesene in *E. coli* [16, 17]. The recently, engineered *Saccharomyces cerevisiae* has produced heterologous artemisinic acid [18, 19], and the *E. coli*-*S. cerevisiae* co-culture system has been engineered to produce oxygenated taxanes and functionalized sesquiterpenes (i.e., nootkatone) [20].

As the MEP pathway exists in the plastid of plants, cyanobacteria, CO_2_-fixing photoautotrophs, have the sole MEP pathway to form terpenoids and precursors of chlorophyll, of which engineering has taken advantage of higher rates of photosynthesis in cyanobacteria than land plants [21]. Most recent metabolic engineering of cyanobacteria has been carried out to directly convert CO_2_ to various biochemicals [22] including isobutyrlaldehyde (1.1 g/L) [23], *n*-butanol (404 mg/L) [24], and 2,3-butanediol (2.3 g/L) [25]. However, the production levels of isoprenoids in engineered cyanobacteria showed significantly low titers when heterologous terpene synthase was expressed only in cyanobacteria: isoprene (approximately, 0.3 mg/L) [26], the monoterpenes limonene (4 mg/L) [27] and *β*-phellandrene (0.07 mg/L) [28], the sesquiterpene bisabolene (0.6 mg/L) [27] and triterpene squalene (0.6 mg/L/OD_730_) [29], and carotenoids (1.1 mg/L/OD_730_) [30]. Because the enzymatic activity of terpene synthase is quite low, an optimized cyanobacterial chassis for isoprenoid production has not been constructed. Also, a majority of cyanobacteria do not produce sesquiterpene and triterpene, although cyanobacterial sesquiterpene synthases have been recently identified in N_2_-fixing filamentous cyanobacteria (*Nostoc punctiforme* PCC 73102 and *Nostoc* sp. Strain PCC 7120) [31]. Thus, a modular metabolic engineering of cyanobacteria is required to provide a synthetic cyanobacterial chassis and to produce high levels of GPP-derived or FPP-derived isoprenoids production, respectively.

In this work, we describe the metabolic engineering of *Synechococcus elongatus* PCC 7942 as a model system with modular expression of the genes encoding the MEP pathway enzymes and terpene phosphate synthases (TPSs). This engineering allowed for the exogenous production of amorpha-4,11-diene and endogenous production of squalene directly from CO_2_.

## Results and discussion

### Designing modularized pathways for the production of amorpha-4,11-diene and squalene

**Fig. 1:**
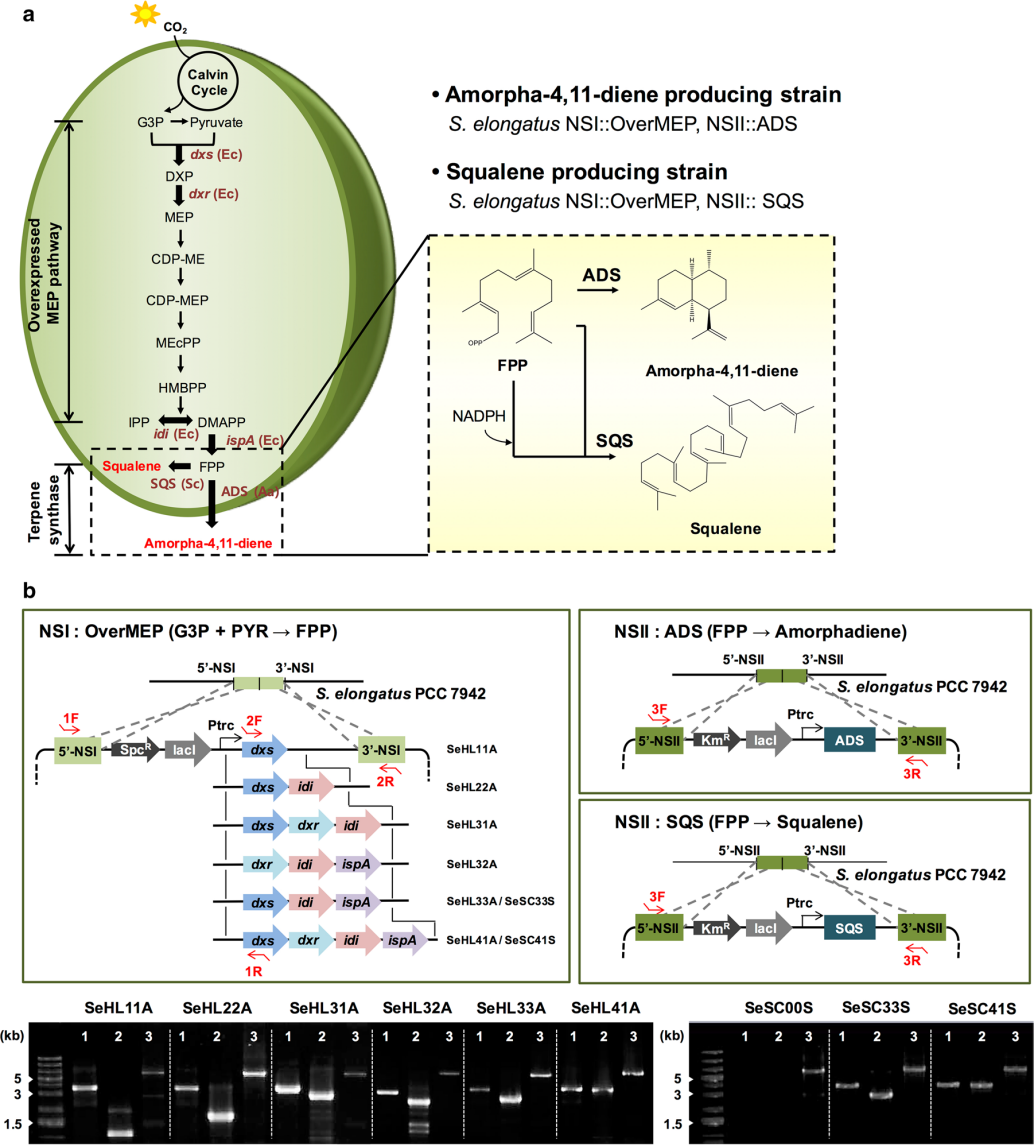
Modular and metabolic engineering of *S. elongatus* PCC 7942 for photosynthetic amorpha-4,11-diene and squalene productions from CO_2_. **a** Heterologous expression of key enzymes derived from *E. coli* were introduced to direct the carbon flux to the native 2-C-methyl-d-erythritol 4-phosphate (MEP) pathway as a module of OverMEP. The second module (*TPS* terpene synthase) is an enzymatic conversion of farnesyl diphosphate (FPP) to either heterologous sesqui- or triterpene as a final carbon sink. Based on the OverMEP strains, heterologous amorphadiene synthase (ADS) and squalene synthase (SQS) were expressed, respectively. A molecule of FPP was converted to amorphadiene catalyzed by ADS, or a reductive dimerization of two molecules of FPP was catalyzed by SQS to produce squalene. **b** Schematic diagrams of the construction of *S. elongatus* PCC 7942 strains that produce either amorphadiene or squalene. The heterologous genes *dxs*, *dxs*-*idi*, *dxs*-*dxr*-*idi*, *dxr*-*idi*-*ispA*, *dxs*-*idi*-*ispA*, *dxs*-*dxr*-*idi*-*ispA* were introduced to a neutral site I (NSI) of *S. elongatus* genomic DNA, respectively. Subsequently, the *ADS* or *SQS* genes were introduced to neutral site II (NSII). Each strain was verified by PCR using a pair of oligonucleotides shown as *red arrows* and their sequences are listed in Additional file 1: Table S1. The corresponding DNA fragments (1, 2 and 3) are shown in gel images. Also, the DNA sequences were verified correctly. Abbreviations of the corresponding gene are *dxs (Ec)* 1-deoxy-d-xylulose-5-phosphate synthase of *E. coli*, *idi (Ec)* isopentenyl diphosphate isomerase of *E. coli*, *dxr (Ec)* 1-deoxy-d-xylulose 5-phosphate reductase of *E. coli*, *ispA (Ec)* farnesyl diphosphate synthase (IspA) of *E. coli*, *ADS (Aa)* amorpha-4,11-dien synthase of *A. annua*, *SQS (Sc)*, squalene synthase of *S. cerevisiae*, *G3P* glyceraldehyde 3-phosphate, *DXP* 1-deoxy-d- xylulose-5-phosphate, *MEP* 2-C-methyl-d-erythritol-4-phosphate, *CDP-ME* 4-diphosphocytidyl-2-C-methyl-d-erythritol; *CDP-MEP* 4-diphosphocytidyl-2C-methyl-d-erythritol-2-phosphate, *MEcPP* 2C-methyl-d-erythritol-2,4-cyclodiphosphate, *HMBPP* (E)-4-hydroxy-3-methylbut-2-enyl-diphosphate, *IPP* isopentenyl diphosphate, *DMAPP* dimethylallyl diphosphate, *FPP* farnesyl diphosphate

*S. elongatus* PCC 7942 possesses the MEP pathway that is used to synthesize isopentenyl diphosphate (IPP) and dimethylallyl diphosphate (DMAPP) from CO_2_. In plants and eukaryotes, biosynthesis of amorpha-4,11-diene and squalene are performed from a substrate FPP or two molecules of FPP, respectively. Thus, we modularized the biosynthesis pathways into two modules (OverMEP and TPS) for optimization [16, 32] (Fig. 1a). First, to increase the intracellular concentration of the FPP substrate, we overexpressed the heterologous genes encoding key enzymes in the MEP pathway and an IspA enzyme of *E. coli* using chromosomal integration vectors (pSe1B1s [33]) targeting neutral site I (NSI) of *S. elongatus* PCC 7942. Four *E. coli* genes (*dxs*, *dxr*, *idi*, *ispA*) documented to increase lycopene [13, 34, 35] were selected and a combinatorial approach of metabolic engineering was applied to identify the bottleneck of the cyanobacterial MEP pathway toward FPP (Fig. 1b).

For the OverMEP modules, overexpressions of the single gene of either the *dxs* (encoding for 1-deoxy-d-xylulose-5-phosphate synthase) or *idi* (encoding for isopentenyl diphosphate isomerase) were tested to confirm if they are good engineering targets as shown previously, resulting in the strains SeHL11A and SeHL12A. In addition, the *dxr* gene (encoding for 1-deoxy-d-xylulose 5-phosphate reductase) and/or *ispA* (encoding for farnesyl diphosphate synthase) genes were chosen for the overexpression in a combination with the *dxs* or/and *idi* genes to investigate whether or not Dxr or IspA is the rate-limiting enzyme in the MEP pathway. As a result, nine recombinant *S. elongatus* strains were generated for the OverMEP modules (Table 1).

**Table 1:** Bacteria strains and plasmids used in this study

Strain or plasmid	Relevant characteristics
Strains	
*E. coli* DH5α [60]	F^−^(80d *lac*Z M15) (*lac*ZYA-*arg*F) U169 *hsd*R17(r^−^ m^+^) *rec*A1 *end*A1 *rel*A1 *deo*R96
Amorphadiene-producing *E. coli* [39]	*E. coli* DH1 harboring pBbA5c-MevT-MBIS plasmid and pADS, Strain 2p
*S. elongatus* PCC 7942	Wild type (ATCC 33912)
SeHL11A	*S. elongatus* NSI::Bb1 s-dxs NSII::Bb2 k-ADS
SeHL12A	*S. elongatus* PCC 7942 NSI::Bb1 s-idi, NSII::Bb2 k-ADS
SeHL21A	*S. elongatus* PCC 7942 NSI::Bb1 s-dxs-dxr NSII::Bb2 k-ADS
SeHL22A	*S. elongatus* NSI::Bb1 s-dxs-idi NSII::Bb2 k-ADS
SeHL23A	*S. elongatus* PCC 7942 NSI::Bb1 s-dxr-idi NSII::Bb2 k-ADS
SeHL31A	*S. elongatus* NSI::Bb1 s-dxs-dxr-idi NSII::Bb2 k-ADS
SeHL32A	*S. elongatus* NSI::Bb1 s-dxr-idi-ispA NSII::Bb2 k-ADS
SeHL33	*S. elongatus* NSI::Bb1 s-dxs-idi-ispA
SeHL33A	*S. elongatus* NSI::Bb1 s-dxs-idi-ispA NSII::Bb2 k-ADS
SeHL41A	*S. elongatus* NSI::Bb1 s-dxs-dxr-idi-ispA NSII::Bb2 k-ADS
SeSC00S	*S. elongatus* NSII::Bb2 k-SQS
SeSC33S	*S. elongatus* NSI::Bb1 s-dxs-idi-ispA NSII::Bb2 k-SQS
SeSC41S	*S. elongatus* NSI::Bb1 s-dxs-dxr-idi-ispA NSII::Bb2 k-SQS
Plasmids^a^	
pSyn_1 (Invitrogen)	pUC, Spc^r^, P_*Ni*_, NSI target sites
pSe1Bb1 s-gfp [33]	pUC, Spc^r^, LacI, P_trc_, BglBrick sites, NSI targeting SyneBrick vector: derived from pSyn_1 vector
pSe2Bb1 k-gfp [33]	pUC, Km^r^, LacI, P_trc_, BglBrick sites, NSII targeting SyneBrick vector
pSe1Bb1 s-dxs	pUC, Spc^r^, LacI, P_trc_, *dxs*(se.co) NSI targeting
pSe1Bb1 s-idi	pUC, Spc^r^, LacI, P_trc_, *idi*(se.co), NSI target site
pSe1Bb1 s-dxs-idi	pUC, Spc^r^, LacI, P_trc_, *dxs*(se.co), *idi*(se.co), NSI targeting
pSe1Bb1 s-dxs-dxr	pUC, Spc^r^, LacI, P_trc_, *dxs*(se.co), *dxr*(se.co), NSI target site
pSe1Bb1 s-dxr-idi	pUC, Spc^r^, LacI, P_trc_, *dxr*(se.co), *idi*(se.co), NSI target site
pSe1Bb1 s-dxs-dxr-idi	pUC, Spc^r^, LacI, P_trc_, *dxs*(se.co), *dxr*(se.co), *idi*(se.co), NSI target site
pSe1Bb1 s-dxr-idi-ispA	pUC, Spc^r^, LacI, P_trc_, *dxr*(se.co), *idi(*se.co), *ispA*(se.co), NSI targeting
pSe1Bb1 s-dxs-idi-ispA	pUC, Spc^r^, LacI, P_trc_, *dxs*(se.co), *idi*(se.co), *ispA*(se.co) NSI targeting
pSe1Bb1 s-dxs-dxr-idi-ispA	pUC, Spc^r^, LacI, P_trc_, *dxs*(se.co), *dxr*(se.co), *idi*(se.co), *ispA*(se.co), NSI targeting
pSe2Bb1 k-ADS	pUC, Km^r^, LacI, P_trc_, *ADS*(se.co), NSII targeting
pSe2Bb1 k-SQS	pUC, Km^r^, LacI, P_trc_, *SQS*(se.co), NSII targeting

^a^ *Km* ^*r*^ kanamycin resistance, *Spc* ^*r*^ spectinomycin resistance, *dxs* (*E. coli*) 1-deoxy-d-xylulose-5-phosphate synthase, *idi* (*E. coli*) isopentenyl diphosphate isomerase, *dxr* 1-deoxy-d-xylulose 5- phosphate reductase; *ispA* (*E. coli*), farnesyl diphosphate synthase (IspA), *ADS* [16] (*A. annua*), amorpha-4,11-diene synthase, *SQS* [36] (*S. cerevisiae*) squalene synthase; (se.co) represents that the gene sequence is codon-optimized to *S. elongatus* PCC 7942. Note that strains and plasmids were constructed in this work unless cited

Next, a heterologous TPS gene was introduced in *S. elongatus* PCC 7942 at neutral site II (NSII) using the second chromosomal integration vector (pSe2B1 k) as a TPS module. For the production of amorpha-4,11-diene and squalene, the *ADS* gene [16] encoding for amorpha-4,11-diene synthase from *A. annua* and the *SQS* gene [36] encoding for squalene synthase from *S. cerevisiae*, respectively, were also inserted into the chromosome. Based on the strains with OverMEP modules, nine and three *S. elongatus* strains were constructed for amorpha-4,11-diene and squalene, respectively (Table 1).

### Cyanobacterial growth for the production of amorpha-4,11-diene from CO_2_ with in situ hexadecane overlay

**Fig. 2:**
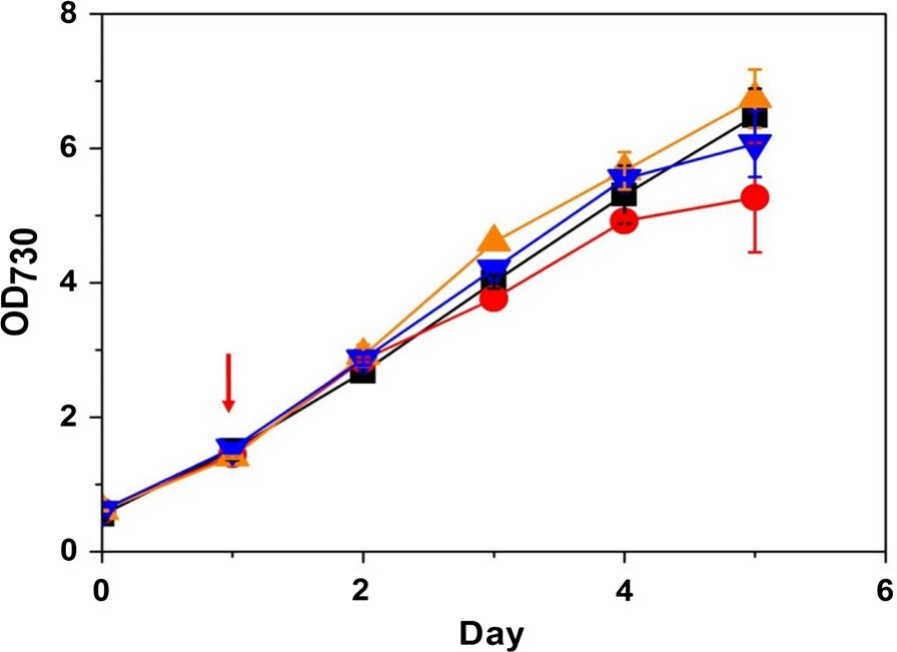
Cellular toxicity of *S. elongatus* PCC 7942 with different solvents for in situ extraction. Growth (OD_730_) of the wild type was measured with different solvents. 20 % (v/v) of the solvent was added into the culture medium at 1 day (indicated in *red arrow*): no solvent overlay (*black square*), dodecane (*red circle*), hexadecane (*orange triangle*), and tetradecane (*blue inverted triangle*). All data are the mean ± standard deviation (SD) from the duplicated cultures

To reduce the cellular toxicity of mono- or sesquiterpenes in microbial cell culture, solvent overlays are applied for in situ extraction [16, 37]. The dodecane overlay has been used to extract limonene and bisabolene from *Synechococcus* sp. PCC 7002 [27]. The relative activity of the cells using a biocompatible organic solvent was correlated with the log P_octanol_, an indicator of the hydrophobicity of the solvent [38]. Here, solvents with log P_octanol_ > 5.5 (dodecane, hexadecane, tetradecane) were selected as a biocompatible organic solvent for *S. elongatus* PCC 7942. Then, the cellular toxicity of each solvent in *S. elongatus* PCC 7942 was investigated by adding 20 % solvent into the culture medium. The growth of *S. elongatus* was slightly inhibited by the addition of dodecane (81.3 % growth of the wild type at 5 days), whereas hexadecane or tetradecane overlay did not inhibit the cell growth (103 or 93.6 %) (Fig. 2). Thus, we selected 20 % (v/v) hexadecane overlay as in situ extraction of for amorpha-4,11-diene.

**Fig. 3:**
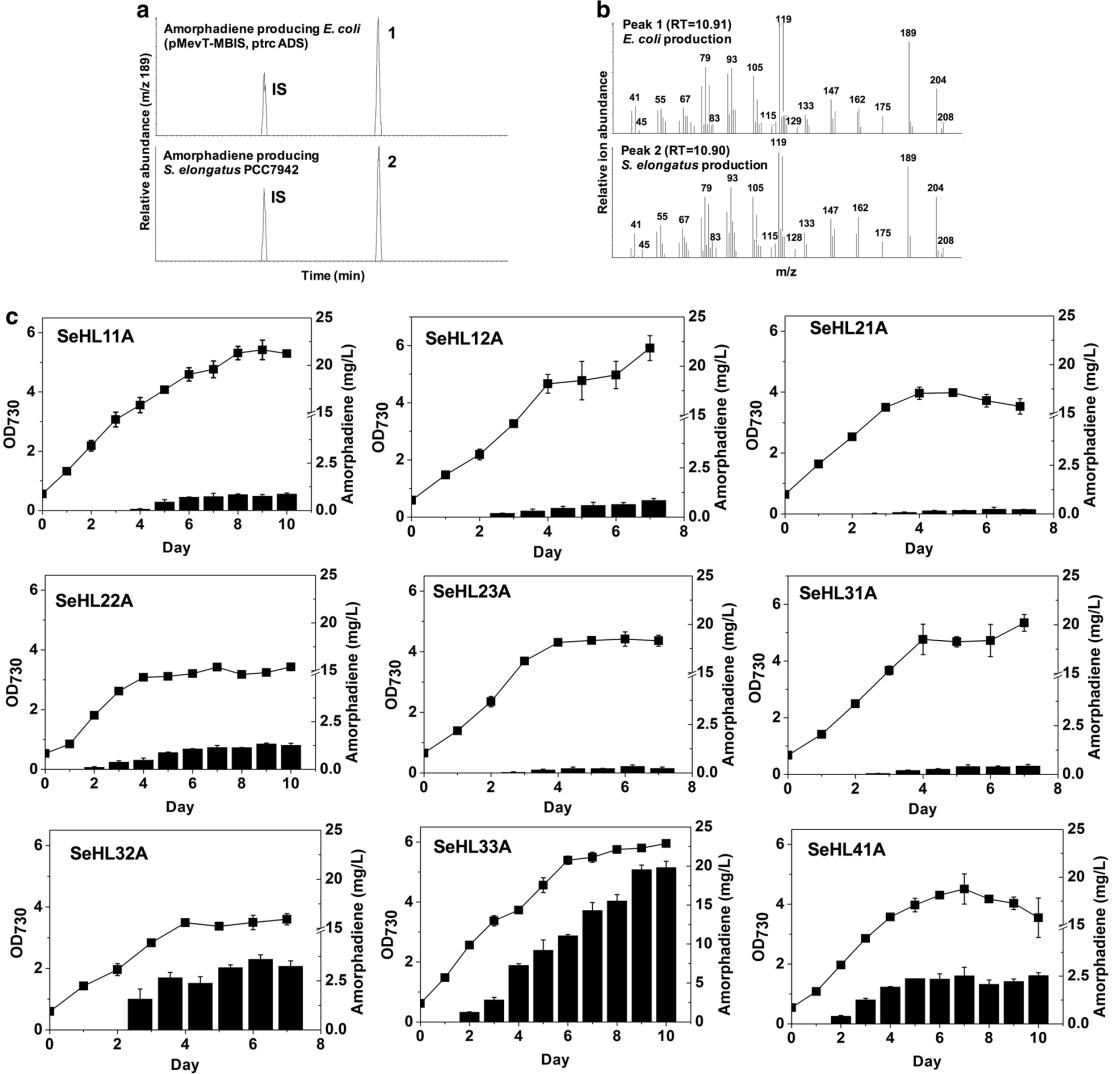
Photosynthetic production of amorpha-4,11-diene from CO_2_ in engineered *S. elongatus* PCC 7942 strains. **a** In situ-extracted samples from hexadecane overlay using engineered *E. coli* strain [39] (*peak 1*) and *S. elongatus* strains (*peak 2*) were analyzed using GC–MS. The internal standard (IS) is caryophyllene. **b** Mass spectra and retention times of amorpha-4,11-diene from either the *E. coli* strain [39] or engineered *S. elongatus* strains*. RT* retention time (in min). **c** Growth (*square*) and production of amorpha-4,11-diene (*bar*) by engineered *S. elongatus* strains: SeHL11A, SeHL22A, SeHL31A, SeHL32A, SeHL33A, and SeHL41A. All data are the mean ± standard deviation (SD) from the triplicated cultures

With the hexadecane overlay, the growth of the recombinant *S. elongatus* strains (SeHL11A, SeHL12A, SeHL31A, SeHL33A) for amorpha-4,11-diene production showed the 18, 8, 17, 18 % growth reductions, respectively, compared to the wild type (Fig. 3). The strain SeHL22A (Dxs-Idi overexpressed) showed severe growth inhibition (less than 48 % growth of the wild type), although a strain with a single overexpression of either *dxs* or *idi* (SeHL11A and SeHL12A) grew better than SeHL22A. Interestingly, strain SeHL21A, SeHL23A SeHL32A, and SeHL41A, where overexpression of Dxr was included, showed severe growth inhibition as well (<54, 66, 55, 54 % growth of the wild type, respectively).

### Photosynthetic production of amorpha-4,11-diene from CO_2_

Using in situ extraction and GC–MS analysis, nine recombinant *S. elongatus* strains with the OverMEP and ADS produced amorpha-4,11-diene that was identical to the amorpha-4,11-diene produced by recombinant *E. coli* strain 2p [39] and secreted into the hexadecane overlay (Fig. 3a, b), whereas the wild type did not produce amorpha-4,11-diene at all. Strain SeHL11A (Dxs overexpressed) and SeHL12A (Idi overexpressed) produced 0.87 mg/L and 0.84 mg/L of amorpha-4,11-diene, respectively. For a combinatorial approach, the co-expression of Dxs and Idi (strain SeHL22A) resulted in an increased level (1.34 mg/L) of amorpha-4,11-diene by 1.54- and 1.59-fold over those produced by strain SeHL11A and SeHL12A, respectively. The next combinatorial engineering was co-expression of Dxr in previously engineered strains. However, co-expression of Dxr with either Dxs or Idi did not increase the levels of amorpha-4,11-diene (SeHL21A and SeHL23A) (Fig. 3c). Strain SeHL31A (Dxs-Dxr-Idi overexpressed) showed even lower levels (0.435 mg/L) than strain SeHL22A by 2.95-fold. Besides Dxr, co-overexpression of IspA was applied to strain SeHL22A and SeHL23A to improve the production levels. As a result, strain SeHL32A (Dxr-Idi-IspA overexpressed) showed 12-fold increased levels of amorpha-4,11-diene (3.59 mg/L) relative to strain SeHL23A (Dxr-Idi overexpressed; 0.3 mg/L of amorpha-4,11-diene). Moreover, strain SeHL33A (Dxs-Idi-IspA overexpressed) showed the significantly increased levels of amorpha-4,11-diene (19.8 mg/L) than SeHL22A (Dxs-Idi overexpressed) by 14.8-fold. As a full construct, SeHL41A (Dxs-Dxr-Idi-IspA overexpressed) showed only 2.54 mg/L of amorpha-4,11-diene, 7.8-fold lower than SeHL33A. Finally, we engineered *S. elongatus* PCC 7942 to produce 19.8 mg/L amorpha-4,11-diene, which is the highest levels among cyanobacterial terpenoids production reported so far.

Based on the results of the production of amorpha-4,11-diene and combinatorial engineering of the MEP pathway, we identified three key factors to produce high levels of amorpha-4,11-diene in *S. elongatus* PCC 7942 using the MEP pathway. First, co-overexpression of Dxs and Idi from *E. coli* in *S. elongatus* PCC 7942 was a good engineering choice to enhance the production of amorpha-4,11-diene from IPP and DMAPP as shown in other studies of isoprenoids [30, 40, 41]. Limonene production [40] (0.27 mg/L for 14 days) was increased by 2.3-fold in *Anavaena* sp. PCC 7120 by co-expressing the limonene synthase (LS) with synthetic Dxs-Idi from *Haematococcus pluvialis* and geranyl diphosphate synthase (GPPS) from *Mycoplasma tuberculosis*, compared to the strain with limonene synthase only. Also, co-expression of the native Dxs-Idi with CrtE (or, GPPS) has led 1.4-fold higher production of limonene (0.05 mg/L/d) in *Synechocystis* sp. PCC 6803 than the parental strain with limonene synthase only.

However, co-expression of Dxr negatively impacted the levels of amorpha-4,11-diene in *S. elongatus* PCC 7942. Engineered strains expressing with Dxr (SeHL21A, SeHL23A, SeHL41A) showed lower levels of both amorpha-4,11-diene production and cell growth than the parental strains (SeHL11A, SeHL12A, SeHL33A), respectively. Strain SeHL31A showed lower levels of amorpha-4,11-diene than the parental strains (SeHL22A). This negative effect has been also observed in *Synechococcus leopoliensis* [42] in which the DMAPP content was not influenced by additional overexpression of Dxr. In addition, overexpression of Dxr has not shown much impact on metabolic balances of the MEP pathway in *Arabidopsis* [43, 44]. As little is known about the multiple levels of the MEP pathway regulation in *S. elongatus* PCC 7942, it is not clear why overexpression of Dxr negatively affected production. Fine-tuning gene expression, such as with the RBS calculator [45] to fine-tune production or by adding protein degradation tags [46] to Dxr to fine-tune degradation, may be required to balance the gene expression of Dxr for the MEP pathway flux, because *E. coli* Dxr has lower *K*_m_ value (30 µM) and faster *k*_*cat*_ (100 s^−1^) than the cyanobacterial enzyme (*K*_m_ of 134 µM and *k*_*cat*_ of 5 s^−1^) [47].

Overexpression of *E. coli*’s IspA was the most crucial factor to increase amorpha-4,11-diene production in *S. elongatus* PCC 7942, where the best producer, strain SeHL33A, showed 22.8-fold higher production than the first engineered strain (SeHL11A) without growth retardation. Whereas *E. coli* has been engineered with IspA for isoprenoid production [17, 35], overexpression of Idi and Dxs was regarded as a key factor to improve production [35, 48, 49]. There have been no FPP synthase activities experimentally verified in *S. elongatus* PCC 7942. Thus, heterologous expression of sesquiterpene synthases without co-expression of FPP synthases in cyanobacteria could produce relatively low amounts of sesquiterpenes [27, 50] compared to the amounts of monoterpenes produced when monoterpene synthases are expressed (i.e., limonene [40, 41]). Overall, multi-omics technologies that integrate metabolomics, proteomics and network-modeling will allow understanding the complex regulation and metabolic aspects including identification of the rate-limiting step in metabolic engineering of cyanobacteria for isoprenoid productions [51]. Furthermore, directed evolution and protein fusions of IspA to GPPS or TPS [17, 52] of *E. coli* could provide a chance to improve production of sesquiterpenes with chain length specificity. Besides IspA of *E. coli*, overexpression of IspA from different sources could be also useful to engineer cyanobacteria.

### Photosynthetic production of squalene from CO_2_

**Fig. 4:**
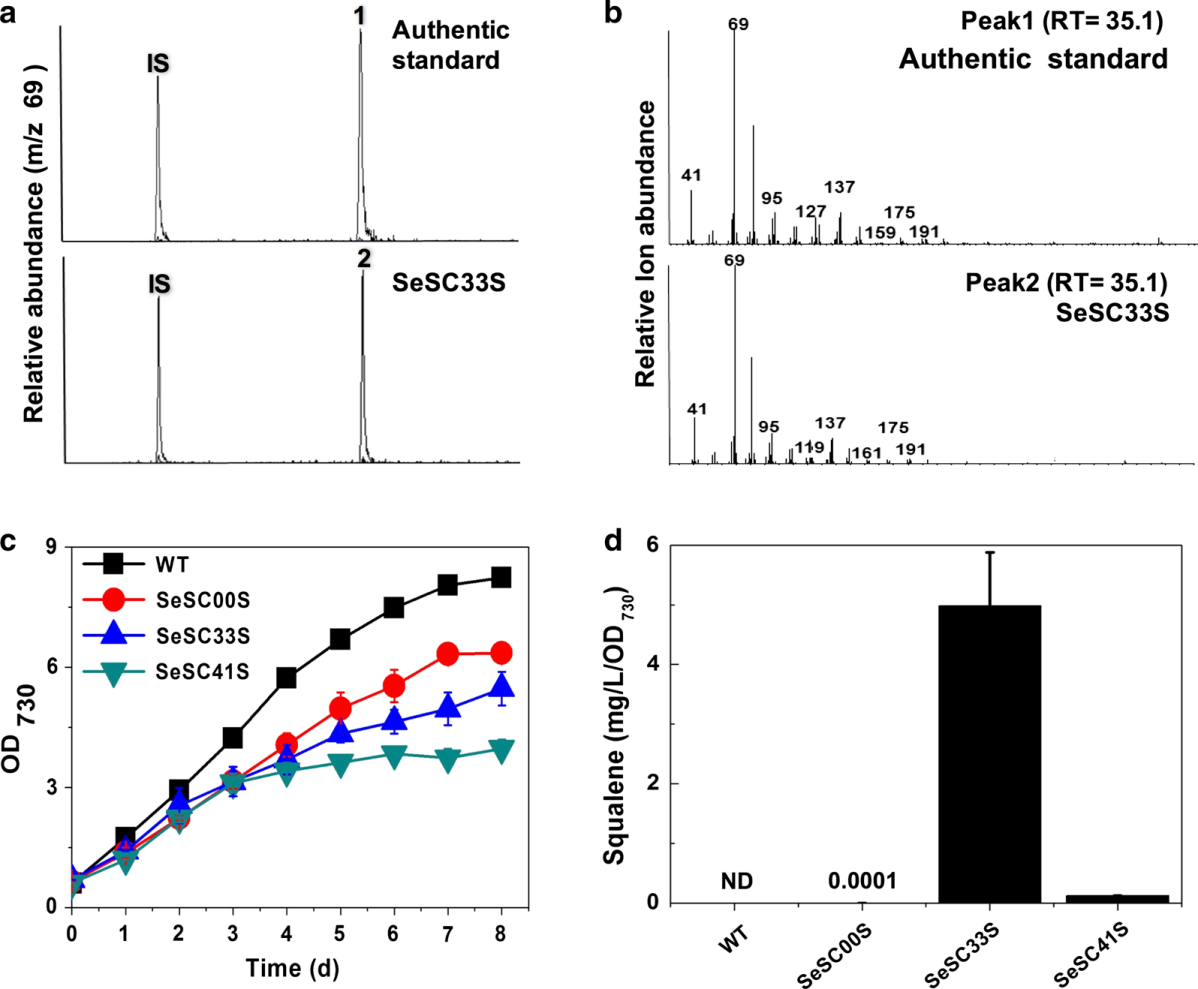
Photosynthetic production of squalene from CO_2_ in engineered *S. elongatus* PCC 7942 strains. **a** Cell pellets from engineered *S. elongatus* strains were extracted using a mixture of chloroform and methanol. Authentic squalene standard (*peak 1*) and extracted samples (*peak 2*) were analyzed with GC–MS. The internal standard (IS) is 1-phenyloctadecane. **b** Mass spectra and retention times of authentic squalene or the samples from engineered *S. elongatus* strains*. RT* retention time (in min). **c** Growth (OD_730_) of wild-type (*black square*) and engineered *S. elongatus* strains: SeSC00S (*red circle*), SeSC33S (*blue triangle*) and SeSC41S (*cyan upper triangle*) strain. **d** Production (mg/L/OD_730_) of squalene from wild-type and the engineered *S. elongatus* SeSC00S, SeSC33S, SeSC41S. All data are the mean ± standard deviation (SD) from the triplicated cultures. *ND* not detected

To determine whether the OverMEP module can be used as a basis for isoprenoid production, we engineered the strain SeHL33 (Dxs-Idi-IspA overexpressed) with co-expression with SQS as another TPS module, since SQS catalyzes the formation of squalene, a triterpene synthesized from two molecules of FPP. Three recombinant *S. elongatus* strains were constructed for the production of squalene (Fig. 4). The wild-type *S. elongatus* PCC 7942 strain did not produce squalene, and a very low amount of squalene (0.0001 mg/L/OD_730_) was produced in a cell by expressing SQS alone (SeSC00S). Similar to SeHL33A, the SeSC33S strain (Dxs-Idi-IspA overexpressed) produced a high amount of squalene in *S. elongatus* PCC 7942 (4.98 mg/L/OD_730_ ±0.90), which was 50,000-fold higher than the strain SeSC00S and the highest levels reported so far in cyanobacteria. In the strain SeSC41S (Dxs, Dxr, Idi, and IspA overexpressed), the squalene content (0.13 mg/L/OD_730_ ±0.01) was reduced by 38.3-fold compared to SeSC33S. The increased or decreased levels were consistent with the previous results of amorpha-4,11-diene production using the SeHL33 or SeHL41 module, respectively. Thus, squalene production studies also support that overexpression of either Dxr was a negative factor or IspA was the most crucial factor in cyanobacteria to increase FPP-derived isoprenoids such as sesquiterpenes and triterpenes along with overexpression of Dxs and Idi, respectively. With respect to the enhanced production of amorpha-4,11-diene and squalene, the SeHL33 with modularized OverMEP proved to be orthogonally functional and expandable to produce other sesquiterpenes or triterpenes with the corresponding TPS’s.

### Alleviating the cyanobacterial growth inhibition by expressing a terpene synthase in *S. elongatus PCC 7942* strain with the optimized MEP pathway only (SeHL33)

**Fig. 5:**
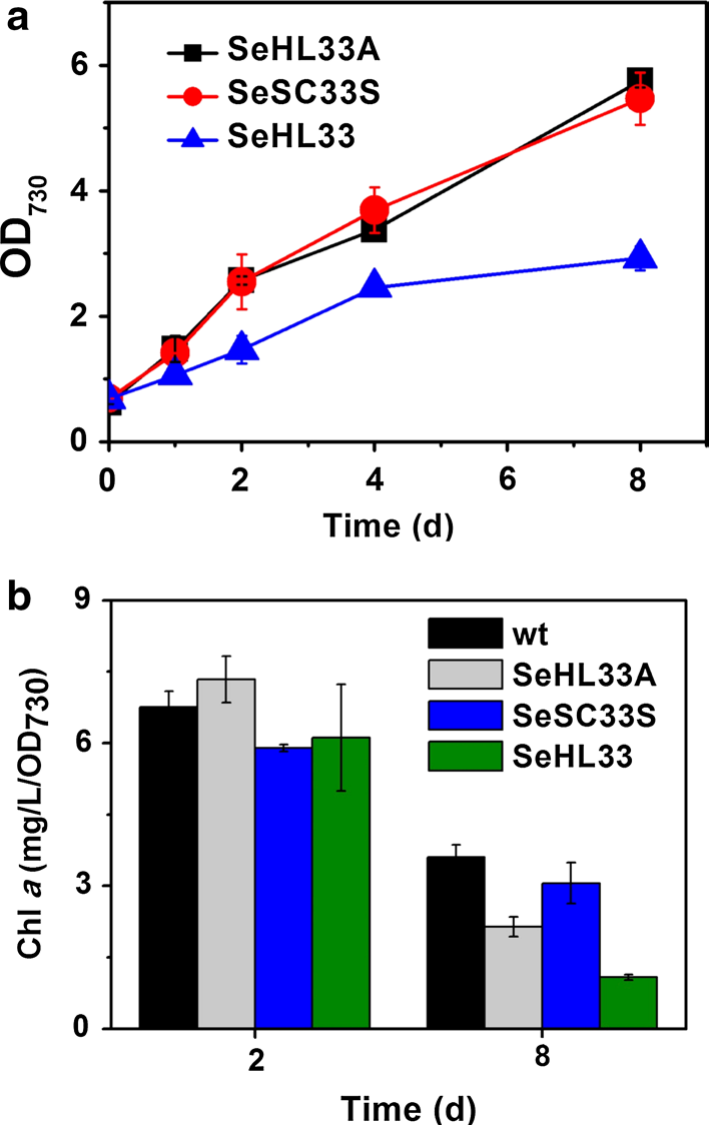
Growth and levels of chlorophyll *a* contents from engineered cyanobacteria. **a** Growth inhibition of the recombinant strains with an OverMEP module, but a TPS. Cyanobacterial cells were cultivated for 7 days with 5 % CO_2_ bubbling. Growth of the strains producing amorphadiene (SeHL33A; *black square*), squalene-producing strain (SeSC33S; *red circle*), and an OverMEP strain lacking a TPS (SeHL33; *blue triangle*) was measured at OD_730_. All data are the mean ± standard deviation (SD) from the triplicated cultures. **b** Levels of Chlorophyll *a* contents (mg/L/OD_730_) from engineered cyanobacteria. Chl *a* contents were measured by a spectrophotometric assay from cyanobacterial cells cultivated for 2 and 8 days. Strains are presented as WT (*black bar*), SeHL33A (*gray bar*), SeSC33S (*blue bar*), and SeHL33 (*green bar*). All data are the mean ± standard deviation (SD) from the triplicated cultures

To expand the FPP-derived isoprenoids production using cyanobacterial strain expressing Dxs, Idi, and IspA (SeHL33), we compared the strain SeHL33 as a basis with amorphadiene-producing SeHL33A and squalene-producing SeSC33S. Previously, the toxicity of FPP, which can accumulate in engineered *E. coli*, has been alleviated by increasing the activity of the terpene cyclase [16]. Thus, we speculated that the strain SeHL33 could also accumulate FPP. In absence of cyanobacterial analysis of proteomics and intracellular FPP accumulation patterns [53], we examined whether or not the strain SeHL33 showed a growth defect due to possible FPP toxicity. As results, the cultures of three recombinant *S. elongatus* strains (strains SeHL33, SeHL33A and SeSC33S) were monitored, showing that the growth of SeHL33 was significantly lower than that of SeHL33A and SeSC33S (Fig. 5a). Moreover, cyanobacterial growth can be measured indirectly using the changes of chlorophyll content [54]. Also, the biosynthesis of Chl *a* is connected to the engineered MEP pathway via geranylgeranyl diphosphate. Chlorophyll *a* contents of the wild-type, SeHL33A, SeSC33S, or SeHL33 strains were determined at 2 and 8 days, resulting in that the Chl *a* content of the strain SeHL33 at 8 days was significantly lower than the Chl *a* contents of other strains (Fig. 5b). Thus, low Chl *a* content (mg/L/OD_730_) of the SeHL33 strain might be related to the growth defect of of SeHL33.

However, our engineered strain SeHL33 with either an ADS or SQS did not show the negative effect on the growth. Thus, alleviation of the cellular toxicity in the engineered MEP pathway with co-expression of terpene synthase in this study was consistent with the previous result in engineered *E. coli* [16]. Furthermore, genome-wide transcriptomics approach could be employed using the strain SeHL33 to increase the levels of isoprenoid production by identifying the stress-response promoters for engineering dynamic pathway regulation [53]. In-depth metabolite analysis (or metabolomics) for the strains could be also needed to understand the bottlenecks and to improve isoprenoid biosynthesis. Since our engineered strains with a modular OverMEP and either an ADS or SQS did not show a negative effect on the growth, we could expand engineering of SeHL33 with different TPSs converting FPP to various sesqui- or triterpenes.

## Conclusion

**Fig. 6:**
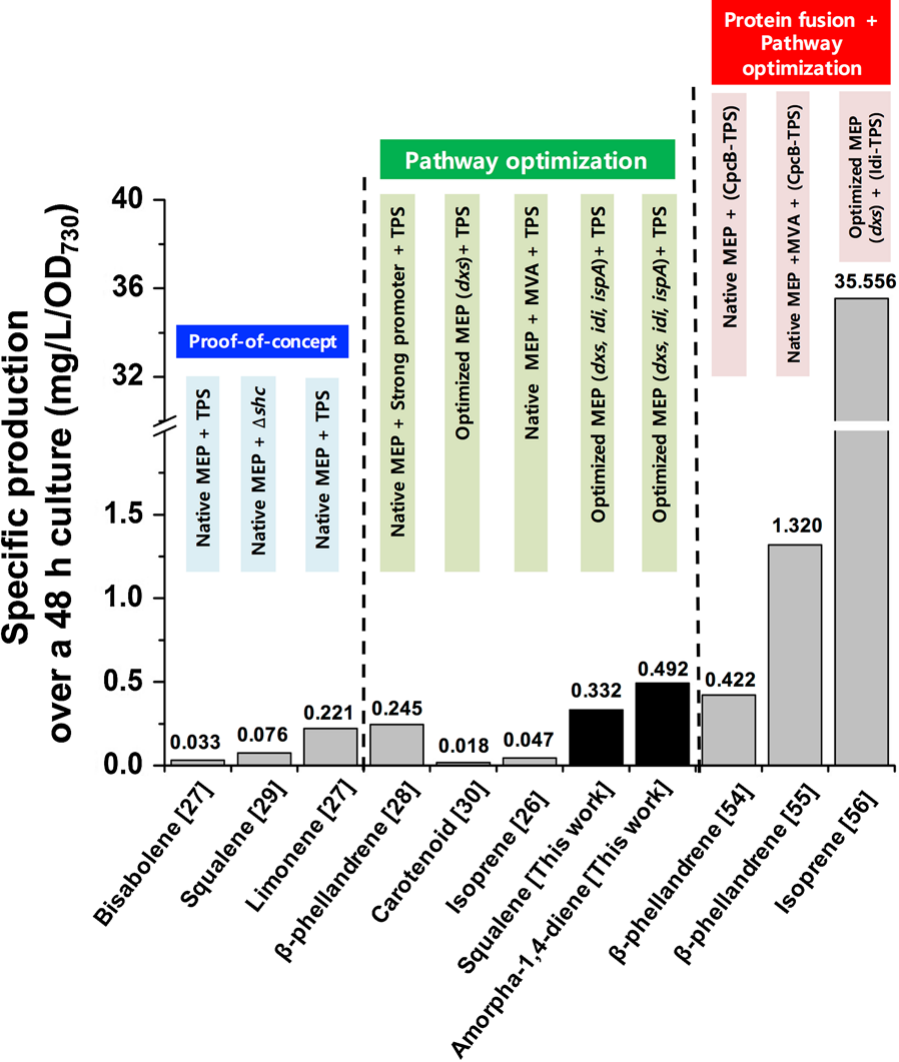
Production comparisons of various isoprenoids from engineered cyanobacteria. Specific production over 48 h culture (mg/L/OD_730_) of amorpha-4,11-diene and squalene (this work in *black bar*) were compared with the previous results (in *gray bar*) as the following: (Group I: the proof of concept; bisabolene from *Synechococcus* sp. PCC 7002 [27], squalene from *Synechocystis* sp. PCC 6803 [29], limonene from *Synechococcus* sp. PCC 7002 [27]) (Group II: Pathway optimization; *β*-phellandrene from *Synechocystis* sp. PCC 6803 [28], carotenoid from *Synechocystis* sp. PCC 6803 [30], isoprene from *Synechocystis* sp. PCC 6803 [26]), and (Group III: Protein fusion with TPS and pathway optimization combined; β-phellandrene from *Synechocystis* sp. PCC 6803 [55, 57], isoprene from *Synechocystis* sp. PCC 6803 [56]). The number on the bar indicates specific production over a 48 h culture. The detailed information is described in “Conclusion” and in the Additional file 1: Table S1. *MVA* Mevalonate

Beyond the proof of concept of isoprenoid productions in cyanobacteria [27, 29], the effort of metabolic engineering of cyanobacteria expanded the list of products from CO_2_ as bio-solar cell factories in this study. Although isoprenoid titers may be influenced by the type of strains, the characteristics of TPS and culture conditions and the high levels of amounts of amorpha-4,11-diene and squalene were produced in this work when pathway optimization was a main strategy of metabolic engineering for isoprenoid production (Fig. 6; Additional file 1: Table S1). Combined with the pathway optimization, increasing the low activity (slow *k*_cat_) of terpene synthase has been shown to be very crucial for the enhanced production of isoprenoid in cyanobacteria [55, 56]. Recently, CpcB as fusion partner or a key MEP enzyme (Idi) was fused to a terpene synthase that resulted in significantly high levels of production of *β*-phellandrene [55, 57] and isoprene [56], respectively. The engineered strains for isoprene production showed the 40 % of photosynthetically fixed carbon fluxes toward isoprenoid biosynthesis pathways. However, there is still much room left for engineering cyanobacteria to improve the titers, compared to other microbial hosts (293 mg/L/OD_600_ of amorphadiene in *E. coli* [58] and 242 mg/L/OD_600_ of amorphadiene in *S. cerevisiae* [18]; 150 mg/L and 55 mg/gDW of squalene in *E. coli* [59]). For example, installing efflux pump protein could improve the amorphadiene in cyanobacteria, of which screening strategy has been successful in *E. coli* [60].

To our knowledge, our optimized modular OverMEP strain (SeHL33) with either co-expression of ADS or SQS demonstrated the highest production levels of amorpha-4,11-diene and squalene from CO_2_.

## Methods

### Chemicals and reagents

All chemicals were purchased from Sigma-Aldrich (St. Louis, MO, USA) unless otherwise specified. Restriction enzymes, Phusion DNA polymerase, and ligases were purchased from Fermentas (Thermo Fisher Scientific Inc., MA, USA).

### Plasmids construction

*E. coli* strain DH5α [61] was used for gene cloning and grown in Luria–Bertani medium (containing per liter: 10 g tryptone, 5 g yeast extract, and 10 g NaCl) at 37 °C, and when appropriate the medium was supplemented with 50 μg/mL kanamycin or 100 μg/mL spectinomycin. All plasmids were derived from SyneBrick expression plasmids (standard vectors for chromosomal integration at the neutral site I (NSI) or II (NSII) using a pSyn_1; pSe1Bb1 s-gfp [33] and pSe2Bb1 k-gfp [33]) using the BglBrick standard cloning method [62] (Table 1). To overexpress key enzymes of the MEP pathway, the *dxs* gene encoding for 1-deoxy-d-xylulose-5-phosphate synthase (Dxs), the *dxr* gene encoding for 1-deoxy-d-xylulose 5- phosphate reductase (Dxr), the *idi* gene encoding for isopentenyl diphosphate isomerase (Idi), and the *ispA* gene encoding for farnesyl diphosphate synthase (IspA) from *E. coli* were codon-optimized to *S. elongatus* PCC 7942 using Gene Designer 2.0 software (DNA2.0, Menlo Park, CA, USA) with a codon usage matrix of *S. elongatus* PCC 7942 and synthesized and cloned into a pSe1Bb1 s vector (targeting at NSI). For production of amorpha-4,11-diene and squalene, the *ADS* gene [16] encoding for amorpha-4,11-diene synthase from *A. annua* and the truncated *SQS* gene [36] encoding for squalene synthase from *S. cerevisiae* were also synthesized and cloned into a pSe2Bb1 k vector (targeting at NSII), respectively.

### Cyanobacterial strain construction and transformation

Transformation of *S. elongatus* PCC 7942 was performed as described previously [63]. The cyanobacterial strains were transformed by incubating cells at a mid-log phase (OD_730_ of 1–2) with 100 ng of plasmid DNA for 24 h in the dark. The mixed culture was then spread on BG-11 plates supplemented with appropriate antibiotics for selection of successful recombination. For selection and culture maintenance, 10 μg/mL spectinomycin or/and 10 μg/mL kanamycin were added into BG-11 agar plates and the BG-11 medium where appropriate. Sub-culture of a single colony was performed to prevent chromosomal segregation. The strains were confirmed by PCR to verify chromosomal integration of targets into either the NSI or NSII (Fig. 1) and the DNA sequences were also correctly verified using a pair of oligonucleotides (Additional file 1: Table S1). *S. elongatus* PCC 7942 strains were constructed by transforming the overexpressed MEP strains with plasmids pSe2Bb1 k-ADS and pSe2Bb1 k-SQS, respectively. Genotypes of recombinant *S. elongatus* strains are listed in Table 1.

### Growth condition for amorphadiene and squalene from engineered cyanobacteria

*S. elongatus* PCC 7942 derivatives for production of amorpha-4,11-diene and squalene were cultivated at 30 °C in the 100 mL culture under continuous fluorescent light (100 µmol photons/m^2^/s) in BG-11 medium supplanted with 10 mM MOPS (pH 8.0). 5 % (v/v) CO_2_ gas was supplied at a constant flow rate of 10 mL/min into the medium. 10 μg/mL spectinomycin or/and 10 μg/mL kanamycin were supplemented for selection pressure. 0.5 mM isopropyl-β-d-1-thiogalactopyranoside (IPTG) was supplemented into the culture medium at 24 h after inoculation for inductions. Especially, 20 % (v/v) hexadecane was added for in situ extraction of amorphadiene.

### Quantification of amorpha-4,11-diene and squalene

Quantification of amorpha-4,11-diene (mg of amorpha-4,11-diene per liter of culture volume) was performed as described previously [16, 64]. The hexadecane layer in the culture was collected and diluted 1:10 with ethyl acetate containing 5 μg/mL of (-)-*trans*-caryophyllene as an internal standard. Hexadecane/ethyl acetates extracts were analyzed using a GC–MS (Agilent 6890 gas chromatograph interfaced with an Agilent 5975 MSD). The extracted sample (1 μL) was injected in split mode (10:1) at an injector temperature of 220 °C and separated using an Ultra-2 capillary column (33 m × 0.2 mm i.d., 0.11 μm film thickness; Agilent Technologies). The initial oven temperature was 100 °C for 3 min and ramped up to 200 °C at 7 °C/min for a total run time of 17.29 min. Helium (99.9999 %) was used as the carrier gas (1.0 mL/min. constant flow at an oven temperature of 150 °C). The ion source temperature was 220 °C. The mass spectrometer was operated at 70 eV in the electron ionization mode with selected ion monitoring (SIM) and the mass range was 35–400 *m/z*. Biosynthetic amorphadiene was obtained from the culture of *E. coli* DH1 harboring the pBbA5c-MevT-MBIS plasmid and pADS (Strain 2p) [39].

For quantification of squalene, the extraction method was performed as described previously [29] with slight modification. 50 mL of the culture was used for extraction. After centrifugation at 3000×*g* for 10 min, the cell pellets were re-suspended with a 2 mL mixture of chloroform and methanol (1:2 ratio) and liquid–liquid extraction occurred for 30 min at room temperature. After additional centrifugation (16,000×*g* for 3 min), the supernatants (200 μL) were collected and the samples supplemented with 20 μg/mL of 1-phenyloctadecane as internal standard were analyzed using a GC–MS. The extracted sample was analyzed using a gas chromatography equipped with a gas chromatography–mass spectrometry (GC-MSD; Agilent Technologies, Santa Clara, CA) equipped with a U2 capillary column (33 m × 0.25 mm, film thickness 0.25 mm, Agilent Technologies, Santa Clara, CA), carrier gas: He (1 mL/min), oven temperature: 90–290 °C (increase rate 6 °C/min). The mass spectrometer was operated at 70 eV in the electron ionization mode with selected ion monitoring (SIM; 69 *m/z*) and mass range was 35–400 *m/z*.

### Measurement of chlorophyll *a*

Cyanobacterial cell cultures (1.5 mL) were harvested and the pigments of cell pellets were extracted in 100 % acetone at 50 °C for 15 min. After centrifugation (16,000×*g* for 3 min), the pigment extract was analyzed for chlorophyll *a* (Chl *a*) by following the previous spectrophotometric assay [65]. The supernatant was obtained and the absorbance was measured at 400–700 nm on Cary 60 UV–Vis spectrophotometer (Agilent technologies, CA, USA).

## Additional file

10.1186/s13068-016-0617-8 Additional data and note for the engineered cyanobacteria strains.

## Abbreviations

GC–MS: gas chromatography–mass spectrometry
FPP: farnesyl diphosphate
MEP: 2-C-methyl-d-erythritol 4-phosphate
GPP: geranyl diphosphate (GPP)
TPS: terpene phosphate synthase
