# Supplementary material for: Photosynthetic conversion of CO2 to farnesyl diphosphate-derived phytochemicals (amorpha-4,11-diene and squalene) by engineered cyanobacteria
Source: Biotechnol Biofuels. 2016 Sep 22;9:202. doi: 10.1186/s13068-016-0617-8 (PMC5034544; doi:10.1186/s13068-016-0617-8)
Supplement: Supplementary file 1 — 10.1186/s13068-016-0617-8 Additional data and note for the engineered cyanobacteria strains. [file 13068_2016_617_MOESM1_ESM.pdf]

Supplementary Table S1. Oligonucleotides used for strain verification in this study.

Primer designation	Relevant characteristics (5’ → 3’)	Source
1F	ACA TCT TCC TGC TCC AGA AG	This study
1R	CTT TTG GGC AGG CAG CCG CTG CTG G	This study
1R’	GCT GGG GCA GGC TCT GAA AG	This study
2F	CCA GCA GCG GCT GCC TGC CCA AAA G	This study
2F’	CTT TCA GAG CCT GCC CCA GC	This study
2R	GAA AGC GTG ACG AGC AGG GA	This study
3F	GGC TAC GGT TCG TAA TGC CA	This study
3R	GAG ATC AGG GCT GTA CTT AC	This study

Note: The strain SeHL32A was verified by primers of 1F and 1R’ and 2F’ and 2R.

Supplementary Notes. Calculation of specific productions for Fig. 6.

[27] Sesquiterpene, bisabolene production from *Synechococcus* sp. PCC 7002

Davies et al. [27] showed that *Synechococcus* sp. PCC 7002 produced 0.3 mg/gDW for 96 h (Conversion factor, OD_{730} 1 = 0.132 gDW/L adapted from the Reference of Wang W, Liu X, Lu X. Engineering cyanobacteria to improve photosynthetic production of alka(e)nes. Biotechnol Biofuels. 2013;6(1):69.). It means that specific production of bisabolene is 0.040 mg/L/OD_{730} (over a 48 h culture is 0.033 mg/L/OD_{730}) and its productivity is 0.006 mg/L/hr.

[29] Triterpene squalene production from *Synechocystis* PCC 6803

Englund et al. [29] showed that specific production of squalene was 0.6 mg/L/OD_{730} in *Synechocystis* PCC 6803 for 192 h (over a 48 h culture is 0.076 mg/L/OD_{730}).

[27] Monoterpene, limonene production from *Synechococcus* sp. PCC 7002

Davies et al. [27] showed that *Synechococcus* sp. PCC 7002 produced 1.7 mg/gDW for 96 h (Conversion factor, OD_{730} 1 = 0.132 gDW/L). It means that specific production of limonene is 0.224 mg/L/OD_{730} (over a 48 h culture is 0.221 mg/L/OD_{730}) and its productivity is 0.031 mg/L/hr.

[28] Monoterpene, β-phellandrene from *Synechocystis* PCC 6803

Formighieri and Melis [28] showed that *Synechocystis* PCC 6803 produced 0.259 mg/gDW for 48 h (Conversion factor, OD_{730} 1 = 0.132 gDW/L). It means that specific production of β-phellandrene is 0.0341 mg/L/OD_{730} (over a 48 h culture is 0.245 mg/L/OD_{730}) and its productivity is 0.00028 mg/L/hr.

[30] Carotenoid production from *Synechocystis* PCC 6803

Kudoh et al. [30] showed that *Synechocystis* PCC 6803 produced 8.4 mg/gDW for 48 h (Conversion factor, OD_{730} 1 = 0.132 gDW/L). It means that specific production of

carotenoids was 1.1 mg/L/OD_{730} in *Synechocystis* PCC 6803 and its productivity is 0.018 mg/L/hr.

[26] Isoprene production of *Synechocystis* PCC 6803

Benently et al. [26] showed that *Synechocystis* PCC 6803 produced 0.25 mg/gDW for 196 h (Conversion factor, OD_{730} 1 = 0.132 gDW/L). It means that specific production of isoprene is 0.033 mg/L/OD_{730} (over a 48 h culture is 0.047 mg/L/OD_{730}) and its productivity is 0.0002 mg/L/hr.

[54] Monoterpene, β-phellandrene from *Synechocystis* PCC 6803

Formighieri and Melis [54] showed that *Synechocystis* PCC 6803 produced 3.2 mg/gDW for 48 h (Conversion factor, OD_{730} 1 = 0.132 gDW/L). It means that specific production of β-phellandrene is 0.422 mg/L/OD_{730} and its productivity is 0.011 mg/L/hr.

[55] Monoterpene, β-phellandrene from *Synechocystis* PCC 6803

Formighieri and Melis [55] showed that *Synechocystis* PCC 6803 produced 10 mg/gDW for 48 h (Conversion factor, OD_{730} 1 = 0.132 gDW/L). It means that specific production of β-phellandrene is 1.32 mg/L/OD_{730} (Incubation not provided).

[56] Isoprene production of *S. elongatus* PCC 7942

Gao et al. [56] showed that *S. elongatus* PCC 7942 produced 525.3 mg/gDW for 504 h (Conversion factor OD_{730} 1 = 0.369 gDW/L [56]). It means that specific production of isoprene is 193.8 mg/L/OD_{730} (over a 48 h culture is 35.55 mg/L/OD_{730}) and its productivity is 2.5 mg/L/hr.
